# Consumption of single cigarettes and quitting behavior: A longitudinal analysis of Mexican smokers

**DOI:** 10.1186/1471-2458-11-134

**Published:** 2011-02-25

**Authors:** James F Thrasher, Victor Villalobos, Joaquin Barnoya, Raul Sansores, Richard O'Connor

**Affiliations:** 1 Arnold School of Public Health, University of South Carolina, Columbia, SC, USA; 2 Instituto Nacional de Salud Pública, Cuernavaca, México; 3 School of Medicine, Washington University in St. Louis, USA; 4 Instituto Nacional de Enfermedades Respiratorias, Mexico City, Mexico; 5 Department of Health Behavior, Roswell Park Cancer Institute, Buffalo, NY, USA

## Abstract

**Background:**

Previous cross-sectional research has suggested single cigarettes could either promote or inhibit consumption. The present study aimed to assess the effects of single cigarette availability and consumption on downstream quit behavior.

**Methods:**

We analyzed population-based, longitudinal data from adult smokers who participated in the 2008 and 2010 administrations of the International Tobacco Control Policy Evaluation Survey in Mexico.

**Results:**

At baseline, 30% of smokers saw single cigarettes for sale on a daily basis, 17% bought singles at their last purchase, and 7% bought singles daily. Smokers who most frequently purchased singles, both in general and specifically to control their consumption, were no more likely to attempt to quit over the 14 month follow-up period than those who did not purchase singles. Frequency of buying singles to reduce consumption had a non-monotonic association with being quit at followup. The odds of being quit was only statistically significant when comparing those who had not bought singles to reduce consumption with those who had done so on a more irregular basis (AOR = 2.30; 95% CI 1.19, 4.45), whereas those who did so more regularly were no more likely to be quit at followup. Frequency of self-reported urges to smoke upon seeing singles for sale was unassociated with either quit attempts or being quit at followup.

**Conclusions:**

These results suggest that the relationship between singles consumption and quit behavior is complex, with no clear evidence that singles either promote or inhibit downstream quit behavior.

## Background

The World Health Organization Framework Convention on Tobacco Control (WHO-FCTC) recommends that the sale of single cigarettes be banned [1,2]. Mexico has banned singles since 1999, before it ratified the WHO-FCTC in 2004. Nevertheless, the sale of single cigarettes appears prevalent in Mexico [3-5], as in other jurisdictions that have banned them [6-10]. The rationale for banning singles primarily reflects concerns about facilitating youth access [11]. However, how adult smokers respond to the availability of single cigarettes is less well understood and merits clarification, especially as the availability and consumption of singles cigarettes could either lower or increase successful smoking cessation.

Access to single cigarettes may promote smoking among adult smokers. For example, because the price of a single cigarette is lower than for a cigarette pack [12-14], smokers who may otherwise quit because of affordability issues may continue to nourish their addiction, albeit while smoking fewer cigarettes. Perhaps reflecting affordability issues, younger smokers and smokers with lower incomes are more likely to smoke singles in Mexico [3]. Furthermore, when a tax increase went into effect in Mexico, consumption went down overall, while the prevalence of singles use increased from 10% to 20% [13]. The visibility of single cigarettes in the surrounding environment may also cue smoking behavior or promote relapse [15]. Indeed, Mexican smokers who experience more frequent cravings to smoke because of seeing singles cigarettes for sale are less likely to intend to quit than Mexican smokers who do not experience such cues or cravings [3]. Similarly, the visibility of singles sales both within and outside of more formal points of sale may support perceptions of the normative nature of smoking. Indeed, the frequency of brand impressions may increase with the visible presence of cigarette packages from which vendors sell singles. Such brand imagery exposures may increase in importance as other advertising channels are banned [16]. Finally, people who consume singles may be less likely to be exposed to health warning labels on cigarette packs compared to people who buy and carry with them the packages that contain these warnings.

The availability of singles cigarettes may also contribute to reductions in cigarette consumption among adults who smoke. In disadvantaged urban settings in the US [14], as well as in Mexico [3], adult smokers report buying single cigarettes as a method to keep consumption down and to quit. Single cigarettes cost around twice as much, per stick, as cigarettes bought in a standard package, so smokers impose upon themselves a steeper monetary cost to limit consumption. Furthermore, purchasing singles imposes greater search costs for each cigarette [17], often involving a substantial increase in the amount of time spent going to the place where single cigarettes are sold, when compared to the time spent reaching into a cigarette package that is kept at hand. In cross-sectional analyses, the frequency of using singles to limit smoking behavior was positively associated with quit intentions among Mexican smokers [3]. Longitudinal analyses are needed to clarify whether the availability of single cigarettes on balance promotes or inhibits smoking among adults.

This study uses longitudinal data from a cohort of smokers representative of six major Mexican cities in order to determine the relationship between consumption of single cigarettes and downstream quit behavior. First, we examine correlates of single cigarette consumption. Second, we examine whether baseline consumption and perceptions of single cigarettes are associated with quit attempts and quitting after baseline.

## Methods

### Study sample

As part of the International Tobacco Control (ITC) Policy Evaluation Survey, data have been collected on an annual basis from adult smokers in Mexico since 2006. The longitudinal analytic sample for the current study consists of data from wave 3 (November to December 2008) and wave 4 (January to February 2010) of the ITC-Mexico survey. Between previous survey administrations, taxes were increased [13], and advertising restrictions and smoke-free policies were implemented [18]; however, no major tobacco control policies were implemented between waves 3 and 4. In six cities (i.e., Mexico City, Monterrey, Guadalajara, Puebla, Tijuana, Mérida) data were collected at both of these waves. A stratified, multi-stage sampling strategy was used within the urban limits designated for each city. Within selected block groups, face-to-face interviews were conducted with randomly selected adult smokers, defined as those who has smoked at least once the previous week and at least 100 lifetime cigarettes (for details, see Thrasher et al., 2009). The wave 3 analytic sample (n = 1649) includes 73% (524/717) of those who were successfully followed up from the wave 2 sample cities (i.e., Mexico City, Guadalajara, Tijuana), of whom 117 were excluded because they had quit by wave 3. The sample also includes a replenishment sample of 203 smokers randomly selected from the same census tracts as those selected for the original sample, as well as new samples of smokers in Mérida, Monterrey and Puebla (n = 813) as well as an augmented sample in Mexico City (n = 135). Household contact and cooperation rates for wave 3 was 79% and 70%, respectively. Sampling weights were developed to account for the likelihood of participant selection. To produce more efficient estimates of association [19], the weights used for model estimation were rescaled to sum to the sample size within each city. The protocol for this study was approved by the IRB at the Mexican National Institute of Public Health. Data are not publically available, but may be requested through http://www.itcproject.org.

### Measures

#### Smoking and quitting behaviour

Baseline (i.e., wave 3) frequency of smoking was used to categorize respondents as nondaily, daily < 5 cigarettes a day or daily > = 5 cigarettes a day. These were dummy coded, with nondaily as the reference group. Baseline intention to quit involved classifying participants as intending to quit in the next 6 months (1) or not (0). Baseline quit behavior was assessed through self-report of attempting to quit in the previous year (1) or not (0). At followup, participants self-reported whether they had attempted to quit in the last year, that is, since wave 3. Participants were also asked at follow up if they continued to smoke or not, and if not, how long they had been quit. Those that had been quit for at least 30 days at follow-up were classified as being quit, as recommended for survey research[20]. A similar period of 4 weeks or more abstinence has been recommended for short clinical trials [21].

#### Singles use and perceptions

At baseline (i.e., wave 3), participants were asked whether their last purchase of cigarettes was a single cigarette, a pack, or a carton of packs, with responses recoded to indicate purchasing either singles (1) or a pack or carton (0). Participants were asked four additional questions about singles, two regarding consumption and two regarding smoking cues. How often did they buy singles and how often did they buy singles to reduce the amount they smoke refer to the former. How often did they see singles being sold and how often did the feel cravings to smoke upon seeing singles being sold refer to the latter. Response options were daily; not daily, but once or more a week; once to three times a month; a few times in the last six months; not in the last 6 months. Due to relatively small sample size in some categories, the first two responses (i.e., daily; not daily, but once or more a week) and the second set of responses (i.e., monthly; a few times in the last six months) were collapsed for analyses where these variables were treated as independent variables. In bi-variate and multivariate analyses, these were dummy coded, with not having exhibited the characteristic in the previous 6 months as the reference group. Interactions among these variables and intentions to quit were assessed by multipliying the ordinal variable by intentions to quit dummy variable.

#### Sociodemographic variables

At baseline (i.e., wave 3), respondents were asked to report their age, sex, highest educational level completed, and monthly income (reported in Mexican pesos; at the time of data collection, $1 USD ≈ $12.7 to $13.6 pesos). Education and income were reclassified to the four categories that reflected the most uniform distribution possible (i.e., less than middle school, middle school, high school or technical school, and more than high school; $0-3000, $3001 to 5000, $5001 to 8000 and $8001 or more pesos per month), and dummy variables were created with the lowest level as the reference group. For income, respondents with missing data were assigned a dummy variable, in order to avoid losing their information in multivariate analyses.

### Analysis

Analyses were conducted using STATA, version 11.0. Univariate descriptions of the study sample and attrition analyses of differences between the sample that was and was not followed up were calculated without taking into account the complex survey design and sampling weights; however, all other analyses took into account the design and weights. Logistic regression was used to estimate crude and adjusted odds ratios that expressed associations between study variables and buying single cigarettes at the last purchase, as well as when determinng the odds of subsequent attempts to quit and being quit. Ordinal regression models were used to estimate the bivariate and multivariate adjusted relationships between study variables and frequency of purchasing single cigarettes. For models that regressed singles purchase behavior on study variables, the baseline (i.e., wave 3) analytic sample of smokers was assessed, whether participants were successfully followed up or not. For logistic models that regressed subsequent quit behavior (i.e., wave 4) on baseline variables (i.e., wave 3), only data were analyzed from participants who were successfully surveyed at both waves.

## Results

Of the smokers who participated in the baseline (i.e., wave 3) survey, 72% (n = 1206/1649) were successfully followed up 14 months year later. Table 1 shows the baseline sample, including characteristics of those who were and were not followed up. Compared to those who were followed up, those who were not followed up were more likely to be male (68% vs. 61%), younger, have higher educational attainment, were less likely to have attempted to quit in the year before baseline (30% vs 35%), and were less likely to have noticed the sale of single cigarettes. Otherwise, the samples were comparable.

**Table 1:** Sample characteristics of Mexican smokers, including those who were and were followed up over 14 months, November/December 2008 to January/February 2010*

Baseline Independent Variables	Baseline (n = 1649)	Followed up	
		Yes (n = 1206)	No (n = 443)
	%(n)	%(n)	%(n)
Sex^b^			
Male	63.0%(1042)	61.0%(739)	68.0%(303)
Age^a^			
18-24	18.5%(305)	18.2%(219)	19.5%(86)
25-39	36.7%(604)	35.2%(425)	40.5%(179)
40-54	28.3%(467)	28.5%(344)	27.8%(123)
55+	16.5%(272)	18.1%(218)	12.2%(54)
Education^c^			
< Middle School	27.3%(443)	29.8%(355)	20.2%(88)
Middle School	29.4%(477)	30.2%(359)	27.1%(118)
Technical School	8.9%(144)	9.3%(111)	7.6%(33)
High School	17.8%(290)	15.7%(187)	23.7%(103)
>High School	16.7%(271)	15%(178)	21.4%(93)
Income (pesos/month)			
0 - 3000	25.2%(412)	25.5%(306)	24.1%(106)
3000 - 5000	24.5%(402)	25.0%(299)	23.4%(103)
5000 - 10 000	19.0%(312)	19.1%(229)	18.9%(83)
8 000 or more	19.4%(317)	18.1%(217)	22.7%(100)
Missing	11.9%(195)	12.3%(147)	10.9%(48)
Smoking intensity			
Non daily	34.0%(560)	33.0%(398)	36.6%(162)
Daily < = 5 cigarettes/day	30.4%(502)	31.0%(374)	28.9%(128)
Daily >5 cigarettes/day	35.6%(587)	36.0%(434)	34.5%(153)
Tried to quit in last year^a^			
No	66.0%(1087)	64.6%(777)	70.0%(310)
Yes	34.0%(559)	35.4%(426)	30.0%(133)
Quit intention in next 6 months			
No	78.5%(1297)	78.8%(950)	78.5%(347)
Yes	21.2%(350)	21.2%(255)	21.5%(95)
Last purchase of cigarettes			
Package	82.1%(1352)	81.3%(980)	84.2%(372)
Singles	16.8%(276)	17.6%(212)	14.5%(64)
Carton	1.0%(16)	1.0%(12)	0.9%(4)
missing	0.2%(3)	0.1%(1)	0.5%(2)
How often bought singles			
daily	7.4%(122)	7.3%(88)	7.7%(34)
not daily but once a week or more	17.3%(285)	16.8%(203)	18.6%(82)
one to three times a month	6.9%(114)	6.8%(82)	7.2%(32)
a few times in the last six months	10.3%(170)	10.3%(124)	10.4%(46)
not in the last 6 months	57.5%(947)	58.3%(703)	55.2%(244)
How often bought single cigarettes to reduce cig consumption			
daily	4.5%(74)	4.3%(52)	5.0%(22)
not daily but once a week or more	12.2%(201)	11.7%(141)	13.6%(60)
one to three times a month	5.6%(92)	5.5%(66)	5.9%(26)
a few times in the last six months	7.2%(118)	6.9%(83)	7.9%(35)
not in the last 6 months	69.7%(1148)	70.7%(853)	66.7%(295)
How often see single cig sold^a^			
daily	29.5%(481)	30.5%(365)	26.7%(116)
not daily but once a week or more	10.7%(174)	11.7%(140)	7.8%(34)
one to three times a month	5.5%(89)	5.6%(67)	5.1%(22)
a few times in the last six months	14.8%(241)	13.5%(162)	18.2%(79)
not in the last 6 months	39.6%(647)	38.7%(463)	42.3%(184)
Feel like smoking when see single cig sold			
daily	18.8%(211)	16.1%(136)	23.2%(75)
not daily but once a week or more	19.3%(225)	19.8%(167)	18.0%(58)
one to three times a month	6.0%(71)	6.3%(53)	5.6%(18)
a few times in the last six months	10.3%(120)	10.5%(89)	9.6%(31)
not in the last 6 months	45.9%(536)	46.9%(396)	43.4%(140)

a: p < 0.05; b: p < 0.01; c: p < 0.001 for comparing those who were and were not followed up.

*Raw estimates, not taking into account complex sample design

Table 2 shows the results of bi-variate and multivariate adjusted logistic regression models, where having purchased singles at last purchase is regressed on the study variables. In bivariate models, higher age and greater household income were associated with lower odds of purchasing singles. However, these associations became non-significant in multivariate models, with the exception of those with middle household income having lower odds of purchasing singles compared to those with the lowest income. Both heavier and lighter daily smokers were less likely than nondaily smokers to have purchased singles, with this association maintaining significance in multivariate models. People who intended to quit in the next six months were more likely than those who did not to have purchased singles in both bivariate (OR = 2.33, 95%CI 1.56, 3.49) and multivariate models (AOR = 2.17, 95%CI 1.45, 3.27). Those who most felt urges to smoke when seeing singles for sale were also much more likely to have purchased singles than those who did not feel these urges.

**Table 2:** Correlates of purchasing singles, adult Mexican smokers

Baseline independent variables		% bought singles at last purchase	Bought singles at last purchase*		Freq of buying singles**		Freq of buying singles to reduce consumption**	
			Bivariate OR (95% CI)	Adj OR*** (95% CI)	Bivariate B (SE)	Adj B*** (SE)	Bivariate B (SE)	Adj B*** (SE)
Entire population		16.5%	--	--	--	--	--	--
Sex	Female	16.5%	1	1	0	0	0	0
	Male	19.3%	1.21[0.90 - 1.61]	1.35[0.96 - 1.91]	0.08[0.11]	0.19[0.14]	-0.03[0.14]	0.06[0.15]
Age	18-24	22.5%	1	1	0	0	0	0
	25-39	20.0%	0.86[0.52 - 1.41]	1.17[0.67 - 2.05]	-0.38[0.19]^a^	-0.24[0.22]	-0.40[0.19]^a^	-0.19[0.23]
	40-55	12.9%	0.51[0.30 - 0.88]^a^	0.87[0.46 - 1.64]	-0.86[0.21]^c^	-0.59[0.24]^a^	-0.75[0.20]^c^	-0.39[0.24]
	55+	17.9%	0.75[0.40 - 1.39]	1.08[0.50 - 2.35]	-1.02[0.25]^c^	-0.93[0.27]^c^	-1.02[0.25]^c^	-0.83[0.33]^a^
Education	< Middle school	21.1%	1	1	0	0	0	0
	Middle school	17.9%	0.82[0.56 - 1.20]	0.63[0.38 - 1.06]	0.05[0.16]	-0.30[0.18]	0.00[0.15]	-0.31[0.21]
	Technical school	18.2%	0.83[0.45 - 1.55]	1.19[0.56 - 2.54]	-0.03[0.22]	-0.05[0.26]	0.10[0.23]	0.14[0.27]
	High school	18.0%	0.82[0.47 - 1.45]	0.66[0.36 - 1.24]	0.03[0.22]	-0.19[0.21]	0.10[0.21]	-0.11[0.27]
	> High school	14.6%	0.64[0.34 - 1.21]	0.7[0.30 - 1.64]	-0.37[0.23]	-0.43[0.25]	-0.20[0.25]	-0.23[0.34]
Monthly household income (pesos)	$0-3000	25.6%	1	1	0	0	0	0
	$ 3001-5000	20.2%	0.73[0.46 - 1.18]	0.89[0.53 - 1.49]	-0.42[0.18]^a^	-0.18[0.18]	-0.30[0.19]	-0.07[0.22]
	$5001-8000	12.6%	0.42[0.27 - 0.65]^c^	0.59[0.36 - 0.95]^a^	-0.89[0.20]^c^	-0.63[0.23]^b^	-0.66[0.22]^b^	-0.27[0.27]
	$8001 or more	15.4%	0.53[0.31 - 0.91]^a^	0.67[0.41 - 1.10]	-0.57[0.22]^a^	-0.21[0.22]	-0.32[0.22]	0.04[0.27]
	Missing	12.9%	0.43[0.24 - 0.77]^b^	0.61[0.29 - 1.30]	-1.15[0.22]^c^	-0.89[0.23]^c^	-0.98[0.26]^c^	-0.62[0.29]^a^
Smoking intensity	Non daily	31.3%	1	1	0	0	0	0
	Daily < = 5 cigs	15.4%	0.40[0.28 - 0.57]^c^	0.41[0.28 - 0.59]^c^	-0.39[0.15]^a^	0.04[0.17]	-0.59[0.17]^c^	-0.31[0.20]
	Daily >5 cigs	7.4%	0.18[0.09 - 0.33]^c^	0.14[0.08 - 0.25]^c^	-0.80[0.16]^c^	-0.58[0.16]^c^	-0.89[0.19]^c^	-0.72[0.20]^c^
Tried quit in last year	No	15.2%	1	1	0	0	0	0
	Yes	23.9%	1.74[1.18 - 2.58]^b^	1.07[0.68 - 1.68]	0.68[0.13]^c^	0.39[0.15]^b^	0.79[0.14]^c^	0.48[0.16]^b^
Intend to quit in next 6 months	No	15.0%	1	1	0	0	0	0
	Yes	29.5%	2.26[1.46 - 3.49]^c^	2.17[1.45 - 3.27]^c^	0.69[0.16]^c^	0.57[0.17]^b^	0.74[0.19]^c^	0.59[0.20]^b^
Urge to smoke when see singles sold	None	8.2%	1	1	0	0	0	0
	3 or less/mo	16.2%	2.17[1.32 - 3.58]^b^	1.84[1.03 - 3.29]^a^	1.45[0.19]^c^	1.36[0.20]^c^	1.67[0.22]^c^	1.59[0.25]^c^
	Weekly or more	39.6%	7.36[5.00 - 10.85]^c^	8.06[5.27 - 12.30]^c^	2.47[0.16]^c^	2.41[0.16]^c^	2.29[0.21]^c^	2.19[0.22]^c^

Observations				1586		1583		1579

a: p < 0.05; b: p < 0.01; c: p < 0.001.

*logistic regression models estimated to determine purchasing singles at last purchase; all variables in models assessed at baseline only.

**ordinal regression model estimated to determine frequency of purchasing singles; all variables in models assessed at baseline only.

***models adjust for all variables shown in the table

Ordinal regression models regressed frequency of purchasing singles on study variables (see Table 2), with results that were generally consistent with models of buying singles at last purchase. In both bivariate and multivariate models, more frequent purchases of singles were made by younger smokers (i.e., 18 to 24 vs. 40 to 54 and vs. 55 and older), lighter smokers (i.e., nondaily vs. daily 5 or more cigarettes a day) and those who intended to quit compared to those who did not. Also, those who were prompted to smoke upon seeing singles for sale more frequently purchased singles, compared to those who did not experience such urges. Bivariate and multivariate models that regressed the frequency of buying singles to reduce consumption on study variables found the same pattern of results for most sociodemographic variables and all smoking-related variables (see Table 2).

Logistic models were estimated to determine whether singles consumption and cueing predicted followup self-report of any quit attempt (see Table 3). Of the variables assessed, only one had a statistically significant association with subsequent quit attempts: those who purchased singles at least once a week to control their consumption had a greater likelihood trying to quit in bivariate models (OR = 1.81; 95% CI 1.31, 2.51), but not in the adjusted models (AOR = 1.56; 95% CI 0.89, 2.74). A series of additional multivariate models were estimated to assess multiplicative interactions between singles consumption variables and other key variables, while adjusting for the same study variables (results not shown). The multiplicative interaction between the dichotomous intention to quit variable and the three level variable of frequency of purchasing singles to control consumption was not statistically significant (p = 0.35). Multiplicative interactions between daily consumption and both the three level purchasing singles to control consumption and the frequency of urges to smoke were not statistically significant (p = 0.31 and 0.08, respectively).

**Table 3:** Predictors of trying to quit during 14 months of followup, adult Mexican smokers*

Baseline independent variables		% tried to quit	Crude OR	Adjusted OR**
Entire population		43.7%	--	--
Frequency of buying singles	None	40.2%	1	1
	3 or less times/mo	46.9%	1.32[0.91 - 1.90]	0.95[0.55 - 1.65]
	Weekly or more	50.0%	1.49[0.98 - 2.26]	0.89[0.45 - 1.76]
Frequency of buying singles to reduce consumption	None	40.7%	1	1
	3 or less times/mo	49.4%	1.42[0.84 - 2.41]	1.45[0.77 - 2.72]
	Weekly or more	55.4%	1.81[1.31 - 2.51]^c^	1.56[0.89 - 2.74]
Urge to smoke when see singles sold	None	42.0%	1	1
	3 or less times/mo	45.0%	1.13[0.67 - 1.92]	0.99[0.58 - 1.70]
	Weekly or more	47.6%	1.26[0.91 - 1.74]	0.98[0.67 - 1.41]

**Observations**				1155

a: p < 0.05; b: p < 0.01; c: p < 0.001.

*logistic regression models estimated; all independent variables were assessed at baseline.

**model adjusts for all variables shown in the table, as well as baseline age, sex, education, income, smoking intensity, previous year quit attempts, and intention to quit.

Logistic models also regressed being quit after 14 months of followup on study variables (see Table 4). In bivariate and adjusted models, frequency of buying singles to reduce consumption predicted being quit, but the association was curvilinear. The increased odds of being quit was only statistically significant when comparing those who had not bought singles to reduce consumption with those who did so at a more irregular basis (AOR = 2.30; 95% CI 1.19, 4.45), whereas those who did so more regularly were no more likely to be quit at followup. In an additional multivariate adjusted model that included an interaction between intention to quit and frequency of purchasing singles to control consumption, the interaction was not statistically significant (p = 0.36). Two additional multivariate adjusted models were run to assess interactions. One model included a multiplicative interaction between daily consumption and the three-level purchasing singles to control consumption variable. The other model included a multiplicative interaction between daily consumption and frequency of urges to smoke. In neither model was the interaction term statistically significant (p = 0.98 and 0.94, respectively).

**Table 4:** Predictors of being quit for 30 days or more after 14 months of followup, adult Mexican smokers*

Baseline independent variables		% quit	Crude OR	Adjusted OR**
			(95% CI)	(95% CI)
Entire population		17.9%	--	--
Frequency of buying singles	None	13.6%	1	1
	3 or less times/mo	20.5%	1.64[1.05 - 2.56]^a^	1.11[0.63 - 1.95]
	Weekly or more	19.3%	1.51[0.99 - 2.32]	1.28[0.70 - 2.37]
Frequency of buying singles to reduce consumption	None	14.3%	1	1
	3 or less times/mo	27.4%	2.26[1.35 - 3.77]^b^	2.30[1.19 - 4.45]^a^
	Weekly or more	18.1%	1.32[0.72 - 2.43]	0.92[0.41 - 2.09]
Urge to smoke when see singles sold	None	15.0%	1	1
	3 or less times/mo	20.8%	1.48[0.81 - 2.70]	1.4[0.76 - 2.58]
	Weekly or more	17.0%	1.15[0.76 - 1.75]	0.92[0.57 - 1.49]

Observations				1155

a: p < 0.05; b: p < 0.01; c: p < 0.001.

*logistic regression models estimated; all independent variables assessed at baseline

**model adjusts for all variables shown in the table, as well as baseline age, sex, education, income, smoking intensity, previous year quit attempts, and intention to quit.

## Discussion

The results from our study indicate that many smokers see and consume single cigarettes in Mexico, in spite of the illegality of their sale. Approximately 30% of smokers saw singles for sale on a daily basis, 18% bought singles at their last purchase, and 31% bought singles in the previous month. These estimates are generally higher than estimates from 2006 [3], although they are not as high as found in a convenience sample of young, disadvantaged adults in the US, where 77% had purchased singles in the previous month [14]. Our results are mostly consistent with the notion that single cigarette use in Mexico is most prevalent among younger smokers and those with lower income and educational achievement. However, associations between singles consumption and these characteristics were somewhat inconsistent across bivariate and multivariate models. More consistent positive associations were found between singles consumption and lighter intensity of cigarette consumption, greater intention to quit, and more frequent urges to smoke upon seeing the sale of singles. This suggests that singles availability may facilitate the early stages of smoking uptake among young people, but they may also maintain low levels of smoking or be used as a method to quit, as has been reported previously [3,14].

Smokers who reported more frequent urges to smoke upon viewing single cigarettes for sale were more likely to purchase singles. However, these same people were no less likely to quit at followup than those who did not report these urges. This lack of association contrasts with cross-sectional research, which found that smokers who reported more frequent urges to smoke because of seeing singles for sale were less likely to intend to quit (AOR = 0.40) [3]. Although our longitudinal results suggest that cueing due to the availability and visibility of singles may not maintain smoking among Mexicans, our self-report measure may not have adequately captured the cueing phenomenon, which can operate at unconscious levels. Cues to smoke have been studied very little in natural settings or through surveys [22], and future research should assess the reliability and validity of self-report and other measures, in order to better understand how cueing works in naturalistic settings. For example, environmental scans indicating widely varying prevalence of singles availability in Mexico [5], could be linked to other data on smoking among people who inhabit these environments.

Our longitudinal results regarding the use of singles as a method to quit were inconsistent. Smokers who frequently purchased singles to control their consumption were no more likely to attempt to quit than those who did not. Estimates of factors that predicted being quit for a month or more produced more inconsistent results, with no increased likelihood of being quit among smokers with the highest frequency of purchasing singles to control consumption; however, less frequent singles consumption was associated with a greater likelihood of being quit at followup (AOR_no urge vs. less frequent urges _= 2.77, 95% CI 1.77, 4.53). When examining either quit attempts or quit success, interactions between intention to quit and the use of singles to reduce consumption were not statistically significant. Hence, it appears that when smokers impose the additional economic costs and search costs of consuming singles as a method to quit, the population-level effect as a harm reduction strategy is unclear. Furthermore, non-statistically significant results around the interactions between consumption intensity and singles consumption suggested that stratification of the data by consumption intensity does not clarify these relationships.

The current study's population-based, longitudinal nature lends strength to these conclusions. However, there were some limitations, including the need for longer studies to better understand relapse, which could be greater in environments with higher prevalence of cues to smoke due to the availability of singles. Furthermore, attrition may have biased results, particularly as the followup sample was slightly more likely to notice singles than those who were not followed up (31% vs. 27% noticing daily). Nevertheless, this difference was not substantial and there were no differences between the analytic sample and those lost to followup on the primary indicators of singles consumption and perceptions. Participation in the study may have been biased in ways that preclude generalization to the sampling frame, although the direction of this bias is not possible to ascertain because of the lack of data on nonparticipants.

The results from this study may not generalize to Mexican populations outside of the sampling frame. However, data were collected in the largest cities in Mexico, and 70% of the Mexican population lives in urban areas [23]. Furthermore, the prevalence of smoking is three times higher in urban areas than in rural areas [24]. Hence, the results likely generalize to the segment of the population that bears a substantial part of the tobacco-related disease burden in Mexico. Similar studies should be conducted with smokers in other settings, including in populations outside of Mexico that have heavier smoking patterns, as the light smoking pattern among Mexicans may restrict these conclusions to Mexico. Finally, a fuller treatment of the implications of singles availability for tobacco control policy development would attempt to address whether smokers who switch from packs to singles would have otherwise quit in the face of interventions, such as tax increases. This type of assessment would likely demand quasiexperimental designs which could compare smoking behavior in countries with different levels of singles availability.

## Conclusions

This study provides the first longitudinal assessment of the relationship between perceptions of single cigarettes, their consumption and quitting behavior. The results suggest that the public health impact of singles consumption among adult smokers in Mexico is unclear. Further studies should more squarely focus on the issue of relapse, switching to singles instead of quitting, and the translatability of this phenomenon to other contexts where single cigarettes are at different levels of prevalence. Without more compelling evidence of their potential to reduce the harms of smoking, countries should be urged to effectively implement and enforce the WHO-FCTC's recommendation to ban singles sales [2].

## Competing interests

The authors declare that they have no competing interests.

## Authors' contributions

JT conceptualized and wrote the majority of the manuscript. VV conducted the statistical analyses and wrote initial drafts of the methods and results. JB, RS and RO provided substantial contributions to the background and implications of the study. All authors read and approved the final manuscript.

## Pre-publication history

The pre-publication history for this paper can be accessed here:

http://www.biomedcentral.com/1471-2458/11/134/prepub
